# Adhesion and host cell modulation: critical pathogenicity determinants of *Bartonella henselae*

**DOI:** 10.1186/1756-3305-4-54

**Published:** 2011-04-13

**Authors:** Bettina Franz, Volkhard AJ Kempf

**Affiliations:** 1 University hospital of the Johann Wolfgang Goethe-University, Institute for Medical Microbiology and Infection Control, Paul-Ehrlich-Strasse 40, Frankfurt am Main, D-60596, Germany

## Abstract

*Bartonella henselae*, the agent of cat scratch disease and the vasculoproliferative disorders bacillary angiomatosis and peliosis hepatis, contains to date two groups of described pathogenicity factors: adhesins and type IV secretion systems. *Bartonella *adhesin A (BadA), the Trw system and possibly filamentous hemagglutinin act as promiscous or specific adhesins, whereas the virulence locus (Vir)B/VirD4 type IV secretion system modulates a variety of host cell functions. BadA mediates bacterial adherence to endothelial cells and extracellular matrix proteins and triggers the induction of angiogenic gene programming. The VirB/VirD4 type IV secretion system is responsible for, e.g., inhibition of host cell apoptosis, bacterial persistence in erythrocytes, and endothelial sprouting. The Trw-conjugation system of *Bartonella *spp. mediates host-specific adherence to erythrocytes. Filamentous hemagglutinins represent additional potential pathogenicity factors which are not yet characterized. The exact molecular functions of these pathogenicity factors and their contribution to an orchestral interplay need to be analyzed to understand *B. henselae *pathogenicity in detail.

## Introduction

*Bartonella henselae *is a gram-negative, zoonotic pathogen with world-wide distribution. Cats have been identified as reservoir hosts but there is evidence that dogs might also serve as a primary reservoir [1,2]. In cats, *B. henselae *causes a long-lasting intraerythrocytic bacteraemia and the organism is transmitted between cats by cat fleas [3,4]. Transmission by other arthropods, such as ticks, has also been suggested [5,6]. Interestingly, the number of reservoir hosts has increased continuously (for overview see [7]).

*B. henselae *is the aetiologic agent of the cat scratch disease (CSD). The organism is transmitted from infected cats to humans by bites or scratches. In most cases, the onset of the disease is a unilateral lymphadenitis in the lymph draining region near the site of the scratch or bite which occurs 2-3 weeks after infection. Usually, CSD is self-limiting and patients do not require antibiotic treatment. However, the clinical course of a *B. henselae *infection can vary from asymptomatic infections towards severe and chronic illness which might cause difficulties in the laboratory diagnosis of such disease. In immunosuppressed patients (e.g., AIDS patients), *B. henselae *infections can result in proliferations of mainly capillary-sized vessels in the skin (bacillary angiomatosis) or liver (peliosis hepatis). The pathogen is detectable within these lesions, and bacterial eradication by antibiotic treatment results in regression of the angiomatous tumours [8,9]. Interestingly, current reports describe that *B. henselae *can also cause bacteremia in immunocompetent patients [10].

Although research with the slow growing and fastidious *B. henselae *is hampered by difficulties in performing molecular genetics and by the lack of suitable animal models, two essential pathogenicity factors of *B. henselae *have been identified and investigated in detail in recent years: (i) *Bartonella *adhesin A (BadA) which mediates adhesion to the extracellular matrix and mammalian host cells and (ii) the VirB/VirD4 type IV secretion system which modulates mammalian host cell functions by injecting *Bartonella *effector proteins (Beps) [11,12]. In addition, a further type IV secretion system (Trw-system), other adhesins and potentially filamentous hemagglutinins might also contribute to the pathogenicity of *B. henselae *[7]. This manuscript gives a brief overview of these pathogenicity factors and their possible interactions in the course of infection.

## *Bartonella *adhesin A (BadA)

**Figure 1:**
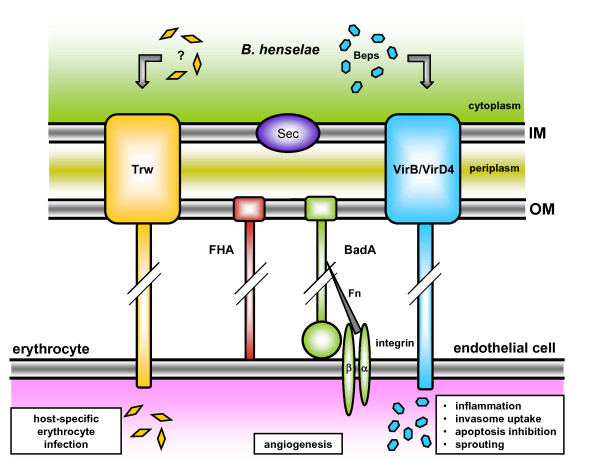
**Possible interactions of confirmed and assumed pathogenicity factors of *B. henselae*: the Trw-system (translocated effectors not known), filamentous hemagglutinin (FHA), BadA and the VirB/VirD4 type IV secretion system (translocated effectors: Beps)**. It can be assumed that BadA (and potentially FHA) ensure a stable contact to the host cell surface for further bacteria-host cell interaction by the type IV secretion system and the Trw system. Abbreviations: IM: inner membrane, OM: outer membrane, Beps: *Bartonella *effector proteins, Fn: fibronectin, BadA: *Bartonella *adhesin A.

Adherence to the host is one of the most important steps during bacterial infection processes. In *B. henselae*, this first and decisive adherence is provided by the trimeric autotransporter adhesin BadA [11] (see Figure 1). Trimeric autotransporter adhesins are widespread in alpha-, beta- and gamma-proteobacteria and play important roles in the pathogenicity of many gram-negative bacteria (e.g., *Yersinia enterocolitica*, *Haemophilus influenzae*, *Moraxella catarrhalis*, *Neisseria meningitidis*) [13,14]. Trimeric autotransporter adhesins are built in a characteristic trimeric, "lollipop"-like surface structure and share a modular organisation consisting of different domains [15]. The C-terminal membrane anchor is homologous throughout all trimeric autotransporter adhesins, forms trimers and provides the autotransport activity. During assembly, trimeric autotransporter adhesins are secreted into the periplasm via the secretory (Sec)-pathway and the membrane anchor forms a pore by building a trimeric 12-stranded beta-barrel in the outer membrane. Head and stalk domains are transported through the pore to the cell surface and the C-terminal part of the stalk locks the pore [16].

Monomeric BadA has a molecular weight of 328 kDa (trimeric: ~ 1.000 kDa) and an enormous length of ~240 nm caused mainly by a long and highly repetitive stalk element. Experiments with a mutant strain lacking almost the whole stalk region suggest that the stalk plays a crucial role in fibronectin binding [17]. Additionally, BadA mediates bacterial autoagglutination, binding to extracellular matrix components (e.g., collagens, laminin), adhesion to host cells and the induction of proangiogenic host cell responses via the activation of hypoxia inducible factor (HIF)-1, the key transcription factor involved in angiogenesis, and the subsequent secretion of angiogenic cytokines [e.g., vascular endothelial growth factor (VEGF)] [11]. These functional properties were assigned to the head domain of BadA which consists of three subdomains: the N-terminal subdomain is homologous to the head of *Y. enterocolitica *adhesin A (YadA) which represents the best characterized trimeric autotransporter adhesin. A highly conserved, short sequence, the neck domain, acts as a linker for two other subdomains (Trp-Ring and GIN) [14,18]. So far, no particular biological functions have been assigned to these subdomains of the BadA head domain.

## Filamentous hemagglutinin

Further to trimeric autotransporter adhesins, other adhesins like filamentous hemagglutinins are known to mediate adhesion to host cells in gram negative bacteria [19,20]. In contrast to trimeric autotransporter adhesins, the presence of filamentous hemagglutinin (FhaB) necessarily depends on a second partner (FhaC/HecB). After Sec-dependent secretion of both proteins to the periplasm, FhaC/HecB forms a transmembrane beta-barrel that allows FhaB to cross the outer membrane. At the cell surface, the protein is further modified and reaches final maturity [21-23].

Analysis of the genome sequence of *B. henselae *revealed eight genes of different lengths encoding homologues of filamentous hemagglutinin (*fhaB1-8*), and four genes encoding homologues of their corresponding partner secretion proteins (*fhaC*/*hecB1-4*) [24]. First and indirect experimental evidence for a potential role of filamentous hemagglutinin in infection process of human endothelial cells derived from a genome-wide transcriptional profiling of a *B. henselae *mutant lacking the response regulator BatR: together with its sensor partner BatS, BatR controlled the expression of horizontally transmitted gene sets critical for the diverse host-associated life styles of *B. henselae*. Expression of *fhaC*/*hecB *was reported to be upregulated by BatR, indicating a possible role of filamentous hemagglutinin in the process of adhesion to host cells [25]. Currently, there are no experimental data but only speculations on the role of filamentous hemagglutinin in the infection process with *B. henselae *[15].

## VirB/VirD4 type IV secretion system

*B. henselae *adhesins might interact with other important pathogenicity factors like type IV secretion systems. Such secretion machineries span the inner and outer membrane of gram-negative bacteria and transfer bacterial effector proteins or DNA to a bacterial or eukaryotic recipient cell probably by a pilus or other surface- protruding filament [26]. The *B. henselae *VirB/VirD4 type IV secretion system (i) mediates rearrangements of the actin cytoskeleton resulting in the formation of bacterial aggregates on the cell surface that are subsequently internalized ("invasome" structure), (ii) triggers a proinflammatory phenotype via activation of nuclear factor (NF)-κB that in turn induces the secretion of interleukin-8 and the expression of the cell adhesion molecules intercellular adhesion molecule (ICAM)-1 and E-selectin and (iii) is crucial for the inhibition of endothelial apoptosis [27]. Moreover, the VirB/VirD4 type IV secretion system is crucially involved in establishing a chronic and bacteraemic infection of the host, as shown in a *Bartonella tribocorum*-rat infection model [12].

To date, seven *Bartonella *effector proteins (BepA-F) have been identified that are translocated by the type IV secretion system machinery [28]. BepA was found to protect endothelial cells from apoptosis [29] and promotes capillary sprouting in an three-dimensional endothelial spheroid infection model. Furthermore, BepG inhibits BepA dependent sprouting and, therefore, both proteins may control angiogenesis during *B. henselae *infections [30]. The combined action of BepC and BepF, but also of BepG itself, induces "invasome"-mediated internalization by inhibiting bacterial endocytosis [31,32]. So far, it can be assumed that the VirB/VirD4 type IV secretion system might interact functionally with BadA and the filamentous hemagglutinin. Potentially, these adhesins establish a first and close contact to the host cell surface and act as spacers providing the optimum distance between the bacterium and the host cell for subsequent protein translocation by the VirB/VirD4 type IV secretion system. Further experiments are required to understand the interaction of these key virulence factors in more detail.

## Trw type IV secretion system

Beside the VirB/VirD4 type IV secretion system, the *B. henselae *genome sequence revealed genes for a second Trw type IV secretion system. Trw-deficient mutants of *Bartonella tribocorum *and *Bartonella birtlesii *were unable to establish a long-lasting bacteremia in their respective animal models. The Trw type IV secretion system was suggested to be essential for intracellular colonization of erythrocytes, whereas a role in infection of endothelial cells was not found [33]. Furthermore, the Trw system mediates host-specificity of erythrocyte infections [34]. Therefore, the Trw type IV secretion system of *B. henselae *might represent a further important pathogenicity factor for establishing chronic bacteremia in the primary (feline) host and possibly interacts with adhesins during the primary infection process, comparable to the VirB/VirD4 type IV secretion system.

## Conclusion

*B. henselae *infections are of increasing interest in veterinary and human medicine. The pathogen causes a long-lasting bacteremia in its feline primary host and cat scratch disease, bacillary angiomatosis, peliosis hepatis and other diseases in humans. Only a limited knowledge about pathomechanisms operating in the course of a *B. henselae *infection exists. The best described pathogenicity factors are the VirB/D4 type IV secretion system, *Bartonella *adhesin A (BadA) and the Trw system. The trimeric autotransporter adhesin BadA ensures the contact of *B. henselae *to host cells and extracellular matrix proteins; filamentous hemagglutinin might also fulfill this function. Adhesion might be a necessary prerequisite to allow subsequent bacterial host cell modulation by the VirB/D4 type IV secretion systems. Currently it is not obvious whether similar pathomechanisms occur in both animal reservoir hosts and human patients. The development of a suitable *B. henselae *animal infection model, especially mimicking vasculoproliferative disorders, would greatly promote *Bartonella *research.

## Competing interests

The authors declare that they have no competing interests.

## Authors' contributions

Both authors conceived the intellectual content and wrote the article. They also read and approved the final manuscript.
